# Perspectives on Epigenetics Alterations Associated with Smoking and Vaping

**DOI:** 10.1093/function/zqab022

**Published:** 2021-04-23

**Authors:** Zidian Xie, Irfan Rahman, Maciej L Goniewicz, Dongmei Li

**Affiliations:** 1 Department of Clinical & Translational Research, University of Rochester Medical Center, Rochester, NY, USA; 2 Department of Environmental Medicine, University of Rochester Medical Center, Rochester, NY, USA; 3 Department of Health Behavior, Roswell Park Comprehensive Cancer Center, Elm and Carlton Streets, Buffalo, NY, USA

**Keywords:** epigenetics, e-cigarette, DNA methylation, miRNA, lncRNA

## Abstract

Epigenetic alterations, including DNA methylation, microRNA, and long noncoding RNA, play important roles in the pathogenesis of numerous respiratory health conditions and diseases. Exposure to tobacco smoking has been found to be associated with epigenetic changes in the respiratory tract. Marketed as a less harmful alternative to combustible cigarettes, electronic cigarette (e-cigarette) has rapidly gained popularity in recent years, especially among youth and young adults. Accumulative evidence from both animal and human studies has shown that e-cigarette use (vaping) is also linked to similar respiratory health conditions as observed with cigarette smoking, including wheezing, asthma, and COPD. This review aims to provide an overview of current studies on associations of smoking and vaping with epigenetic alterations in respiratory cells and provide future research directions in epigenetic studies related to vaping.

## Introduction

Electronic cigarette (e-cigarette) is a battery-operated device that heats a liquid and allows users to inhale an aerosol, which usually contains nicotine, ultrafine particles, flavorings such as cytotoxic cinnamaldehyde and diacetyl (a chemical linked to a serious lung disease), volatile organic compounds such as respiratory irritants acrolein and acrylamide, lung cancer-causing chemicals such as formaldehyde and acetaldehyde, and heavy metals such as nickel, tin, and lead.^1–4^ Those respiratory toxicants and irritants present in aerosols generated from e-cigarettes are delivered to lungs with every puff taken by e-cigarette user. E-cigarettes have rapidly gained popularity in the United States in recent several years, especially among youth and young adults.^1^^,^^5^ E-cigarettes use (vaping) in youth has also been shown to be associated with subsequent cigarette smoking.^6^^,^^7^ Using large national survey data, our epidemiology studies have found the association of vaping with self-reported wheezing and chronic obstructive pulmonary disease (COPD) in US adults,^8–10^ and the association of vaping with self-reported difficulty concentrating, remembering, or making decisions in both US youth and adults.^11^^,^^12^ Our recent findings that e-cigarette aerosols cause oxidative stress, DNA damage, and inflammatory responses in human lung epithelial cells and mouse lungs indicated respiratory disease risk associated with e-cigarette use, which is consistent with previous studies.^13–18^ While flavoring chemicals are commonly used in e-cigarettes, there is limited information on the adverse health effects of those flavorings in e-cigarettes.^19^ We have shown that e-cigarette flavoring chemicals such as acetoin, ortho-vanillin, and maltol can stimulate the release of IL-8 in human bronchial epithelial cells (Beas2B) and human lung fibroblasts (HFL-1) to trigger inflammatory responses.^14^^,^^20^ A recent review of toxicological effects of tobacco and menthol-flavored e-cigarettes summarized the cytotoxicity and genotoxicity of tobacco and menthol-flavored e-cigarette inhalation exposure from both mice and human cell line studies, including increased oxidative stress, apoptosis, inflammation, DNA damage, and epithelial barrier dysfunction.^21^

Epigenetic alterations, including DNA methylation changes, dysregulation of microRNAs (miRNAs), and long noncoding RNAs (lncRNAs), have been found to play some important roles in the initiation and development of human diseases, as well as the interactions between genetic and environmental factors.^22–33^ DNA methylation is an epigenetic mechanism involving transferring a methyl group to the C5 position of the cytosine to form 5-methylcytosine, which could regulate gene expression.^34^ DNA methylation has been involved in autoimmune diseases, metabolic disorders, neurological disorders, and the aging process.^35^ miRNAs are a class of small noncoding RNAs with 21–25 nucleotides that play important regulatory roles in a wide range of cellular and biological processes such as immune regulation and inflammatory response.^36–39^ miRNAs have been reported to play crucial roles in pathophysiology of chronic inflammatory lung diseases and lung cancers.^40–43^ lncRNAs are a type of noncoding RNAs with more than 200 nucleotides in length.^44–46^ lncRNAs are a relative abundant component of transcriptome, which have been identified to have important cellular functions including regulation of gene transcription, cell differentiation, cancer cell invasion, and metastasis, and chromatin remodeling.^47–50^ Increasing evidence showed that lncRNAs regulate many physiological and pathological responses, including immune cell differentiation and activation, metabolism and glucose homeostasis, cardiovascular development, brain temporal and spatial expression patterns, and responses to environmental exposures. Deregulation of lncRNA is responsible for numerous diseases in mammals, and lncRNA has shown their significance as biomarkers in cancer prognosis and diagnosis.^49–53^

## Epigenetic Alterations Associated with Smoking

Cigarette smoking is a well-known risk factor for cancer, cardiovascular disease, and COPD.^54^ Smoking exposure was considered a strong environmental modifier and has been found to be associated with epigenetic changes across tissue types in several studies.^55–58^ Smoking has been shown to modulate DNA methyltransferase 1 (DNMT1) and histone modification enzymes that are involved in pathogenesis of lung cancer and COPD.^59^ A previous metaanalysis of genome-wide DNA methylation studies found the association of smoking with DNA methylation changes (2623 CpG sites) linked with pulmonary functions, cancers, inflammatory diseases, and heart disease.^60^ Another study showed that smoking-associated DNA methylation biomarkers had a strong association with cognitive function, brain structure, physical health, and psychosocial health.^58^ Investigating the DNA methylation alterations associated with smoking not only helps us understand the mechanisms in pathogenesis of those diseases associated with smoking exposure, but also identifies biomarkers used for cigarette consumption prediction. For example, DNA methylation status at locus cg05575921 examined either in human whole blood or saliva samples could be used as a biomarker to differentiate smokers from nonsmokers as well as a predictor for daily cigarette consumption.^61^^,^^62^

In addition to DNA methylation alterations, smoking exposure can lead to noncoding RNAs change such as miRNAs and lncRNAs. Using whole blood samples from Framingham Heart Study participants, a six-miRNA signature of smoking was found to be associated with smoking-induced inflammation and reduced pulmonary functions.^63^ A genome-wide lncRNA expression in human lung tissue study showed that hundreds of lncRNAs were differentially expressed between five smokers with COPD, five smokers without COPD, and three nonsmokers without COPD, which suggest that smoking can change the expression of many lncRNAs.^64^ Gene enrichment analysis of identified significant lncRNAs showed changes in key pathogenic processes of COPD due to smoking.^65^ In vitro human bronchial epithelial (HBE) cell studies indicated the involvement of lncRNAs in the epithelial–mesenchymal transition and malignant transformation of the HBE cells induced by cigarette smoke extract.^66^

## Epigenetic Alterations Associated with Vaping

Very few studies have investigated the epigenetic changes associated with vaping, and the potential association of DNA methylations, miRNAs, and lncRNAs with the health effects of vaping (Table 1). Previous mouse studies showed that maternal e-cigarette exposure could lead to global DNA methylation changes and cognitive problems such as deficits in short-term memory, reduced anxiety, and hyperactivity in the offspring.^67^^,^^68^ Using 45 human peripheral blood samples from exclusive vapers, smokers, and controls (nonusers), recent study showed significantly reduced methylation levels in LINE-1 repeat elements and global DNA hydroxymethylation in both vapers and smokers compared with controls.^69^ Meanwhile, no significant difference in those DNA methylation levels was observed between exclusive vapers and smokers.^69^

**Table 1.** Current Literature on Epigenetic Alterations Associated with Vaping

Epigenetic alterations	Study sample	Tissue	Groups	Sample size	Study results	Reference
DNA methylation	Mice	Brain	Ambient air E-cigarette aerosols with nicotine E-cigarette aerosols without nicotine	8 in each group	Global DNA methylation was significantly increased in Group 3 compared to Group 1. No significant change between Group 1 and Group 2.	Nguyen et al.^67^
DNA methylation	Mice	Lung	Ambient air E-cigarette aerosols with nicotine E-cigarette aerosols without nicotine	3 in each group	Global DNA methylation was significantly increased in Groups 2 and 3 compared to Group 1.	Chen et al.^68^
DNA methylation	Human	Peripheral blood	Vapers Nonsmokers and nonvapers	15 in each group	Demethylation in the LINE-1 repeat elements and decreased global methylation was significant between groups.	Caliri et al.^69^
Exosomal miRNAs	Human	Plasma	Vapers Nonsmokers	7 vapers and 8 nonsmokers	13 upregulated and 4 downregulated miRNAs were significant between groups.	Singh et al.^70^
Exosomal lncRNAs	Human	Plasma	Vapers Nonsmokers	6 in each group	13 upregulated and 5 downregulated lncRNAs were significant between groups	Kaur et al.^71^

Exosomes have been reported recently to mediate cell-to-cell communication and affect many physiological processes.^72–75^ Exosomes are small nano-sized vesicles released by different cell types such as immune and structure cells.^76^ Exosomes contain enriched amount of surface proteins, regulatory proteins, mRNAs, miRNAs, and lncRNAs.^77^ Our recent study using plasma exosomes from seven cigarette smokers, seven vapers, and eight nonsmokers identified 24 significant miRNAs between smokers and nonsmokers, and 17 significant miRNAs between vapers and nonsmokers.^70^ Examination of the 24 miRNAs and 17 miRNAs showed 9 overlapped miRNAs, which indicated both similarities and differences in the miRNA perturbations between smoking and vaping.^70^ Identified miRNAs have been found to be involved in multiple biological pathways and processes in respiratory tract, such as regulation of nucleotide and nucleic acid metabolism and transcription factor activities using functional enrichment analysis. One of the identified significant miRNA hsa-let-7a-5p was found to be able to differentiate nonsmokers from tobacco users.^70^ Another study by our group using human plasma exosomes from six smokers, six vapers, and six nonsmokers identified seven significant lncRNAs between smokers and nonsmokers, 13 significant lncRNAs between vapers and nonsmokers.^71^ Examination of the 7 lncRNAs and 13 lncRNAs did not show overlapped lncRNAs, which indicated the differences in the lncRNA perturbations between smoking and vaping.^71^ Functional analysis of identified lncRNAs showed the involvement of those significant lncRNAs in the biological process such as steroid metabolism, hemopoiesis, and regulation of cell proliferation.^71^

## Conclusions and Future Perspectives

Emerging evidences from mice and human studies suggest potential association of vaping with epigenetic alterations. Given the similarities (eg, nicotine) and differences (eg, combustion byproducts) in chemical compositions between tobacco smoke and e-cigarette aerosols, our recent studies found both common and different epigenetic changes when comparing smokers and vapers with controls.^70^^,^^71^ How these epigenetic alterations could assist us to understand relative risks of vaping compared to smoking is still unclear. Meanwhile, whether the differences in the epigenetic changes between vapers and cigarette smokers could result in toxicities unique to vaping such as the EVALI (e-cigarette, or vaping, product use-associated lung injury) cases in vapers awaits further investigation.^78^ However, the role of those identified epigenetic biomarkers in the etiology of vaping-associated diseases remains unanswered. All of current studies are cross-sectional. Thus, within-subject epigenetic alterations during the e-cigarette initiation and cessation process warrant further investigation. Meanwhile, how the epigenetic level changes when e-cigarette users switch flavors such as switching from fruit flavor (mainly including maltol and furaneol flavoring chemicals) to menthol (L-menthol flavoring chemicals) or tobacco (mainly 2,3,5-trimethylpyrazine flavoring chemicals) flavor in response to the US Food and Drug Administration (FDA) flavor enforcement policy are unknown, which need to be investigated in the future.

With the development of new methods and technologies in epigenetic studies, the epigenetic changes could be examined in an increasingly higher resolution to allow new discoveries in epigenetic changes associated with smoking and vaping. For example, the Perturb-ATAC approach that combines multiplexed CRISPR technique with chromatin accessibility analysis within a single cell to determine the role of transregulatory factors, the new method of selecting DNA methylation-based biomarkers through different biophysical properties to distinguish cancer cells from noncancerous cells, and a multiplexed mass cytometry assay to investigate the global levels of 40 different histone modifications at single-cell resolution.^79–81^ Meanwhile, studies on cell-type-specific and tissue-specific epigenetic changes will allow us to have a better and deeper understanding of the role of epigenetic changes plays in the etiology of disease development. These new technologies on epigenetic studies as well as tissue and cell-specific epigenetic studies could facilitate us to understand how smoking and vaping affect the epigenetic changes in different cell or tissue types, and potential health risks associated with them. Scientific endeavors in terms of understanding the epigenetic/epigenomic changes based on multiomics and spatial transcriptomics will help determine the toxicities of vaping at cellular/subcellular levels.

## Funding

Research reported in this publication was supported by the National Cancer Institute of the National Institutes of Health (NIH) and the Food and Drug Administration (FDA) Center for Tobacco Products under Award Number U54CA228110. The content is solely the responsibility of the authors and does not necessarily represent the official views of the NIH or the Food and Drug Administration (FDA).

## Conflict of Interest Statement

M.L.G. reports research grant from Pfizer and personal fees from Johnson & Johnson, outside this work. Other authors have no potential conflict of interest to declare.

## Authors’ Contributions

Z.X., I.R., M.L.G., and D.L. conceived and designed the study; Z.X. and D.L. wrote the original manuscript; Z.X., I.R., M.L.G., and D.L. contributed to editing the manuscript.
